# Isolation of brain mitochondria from neonatal mice

**DOI:** 10.1111/j.1471-4159.2011.07525.x

**Published:** 2011-11-03

**Authors:** Xiaoyang Wang, Anna-Lena Leverin, Wei Han, Changlian Zhu, Bengt R Johansson, Etienne Jacotot, Vadim S Ten, Neil R Sims, Henrik Hagberg

**Affiliations:** * Perinatal Center, Institute of Neuroscience and Physiology, University of GothenburgGothenburg, Sweden; † Department of Pediatrics, The Third Affiliated Hospital of Zhengzhou UniversityZhengzhou, China; ‡ Center for Brain Repair and Rehabilitation, HInstitute of Neuroscience and Physiology, University of GothenburgGothenburg, Sweden; § The Electron Microscopy Unit, Institute for Biomedicine, University of GothenburgGothenburg, Sweden; ¶ Institute of Reproductive and Developmental Biology, Imperial CollegeLondon, UK; ** Inserm U676, Hôpital Robert DebréParis, France; †† Université Paris DiderotUMR676, Paris, France; ‡‡ Departments of Pediatrics, Columbia UniversityNew York, NewYork, USA; §§ Centre for Neuroscience and Discipline of Medical Biochemistry, School of Medicine, Flinders UniversityAdelaide, South Australia, Australia; ¶¶ Perinatal Center, Department. of Obstetrics and Gynecology, Sahlgrenska AcademyGothenburg, Sweden

**Keywords:** brain, mitochondrial isolation, mouse, neonatal

## Abstract

Mitochondria are key contributors to many forms of cell death including those resulting from neonatal hypoxic-ischemic brain injury. Mice have become increasingly popular in studies of brain injury, but there are few reports evaluating mitochondrial isolation procedures for the neonatal mouse brain. Using evaluation of respiratory activity, marker enzymes, western blotting and electron microscopy, we have compared a previously published procedure for isolating mitochondria from neonatal mouse brain (method A) with procedures adapted from those for adult rats (method B) and neonatal rats (method C). All three procedures use Percoll density gradient centrifugation as a key step in the isolation but differ in many aspects of the fractionation procedure and the solutions used during fractionation. Methods A and B both produced highly enriched fractions of well-coupled mitochondria with high rates of respiratory activity. The fraction from method C exhibited less preservation of respiratory properties and was more contaminated with other subcellular components. Method A offers the advantage of being more rapid and producing larger mitochondrial yields making it useful for routine applications. However, method B produced mitochondria that were less contaminated with synaptosomes and associated cytosolic components that suits studies that have a requirement for higher mitochondrial purification.

Mitochondria play an important role in development and cell death in different forms of brain injury including neonatal hypoxic-ischemic brain injury (Toman and Fiskum 2011; Hagberg *et al.* 2009; Wang *et al.* 2009) (Blomgren *et al.* 2003). Because of limited cost and the possibility of genetic manipulation, mice have gained popularity for experimental studies of perinatal brain injury. However, there is no widely accepted method for mitochondrial isolation from the neonatal mouse brain even though good methods for mitochondrial isolation have been successfully established for adult rat brain (Sims and Anderson 2008). Methodologies have been applied for mitochondrial isolation from the neonatal brain in rats (Rajapakse *et al.* 2001; Trichet *et al.* 2008) and in mice (Caspersen *et al.* 2008) but, the advantages and disadvantages of these procedures are unknown and it is still unclear which method is preferable. Here we compared minor modifications of three widely used brain mitochondrial isolation methods from the literature (Sims 1990; Rajapakse *et al.* 2001; Hansson *et al.* 2003; Caspersen *et al.* 2008; Sims and Anderson 2008; Trichet *et al.* 2008).

The procedure previously reported for neonatal mice produced mitochondria with well-preserved respiratory properties (Caspersen *et al.* 2008). However, this report provided little information on mitochondrial yield or on the extent of residual contamination. The authors reported that contamination with synaptosomes was observable in electron micrographs of the mitochondrial fraction. Additional approaches, such as the assay of enzyme markers that could have provided a more readily quantifiable assessment of the extent of contamination, were not reported. In the present study, this protocol has been slightly modified (method A) and compared with adaptations of two additional procedures that were thought likely to provide useful alternatives for isolating mitochondria from neonatal mouse. One of these methods (method B) is a minor modification of protocols that have been widely used for preparing mitochondria from the brains of adult rats and from a range of other species (Sims 1990; Rajapakse *et al.* 2001; Hansson *et al.* 2004; Sims and Anderson 2008). The modification involved the use of 19% Percoll instead of 26% in the middle layer during gradient centrifugation as this provided better recovery of neonatal mitochondria in preliminary studies. The other procedure (method C) combined two previous protocols for neonatal rodent brain mitochondrial isolation (Rajapakse *et al.* 2001; Caspersen *et al.* 2008), using combined two successive differential centrifugations and an isopycnic centrifugation but a two-phase Percoll gradient (12% and 26% Percoll) (Rajapakse *et al.* 2001; Caspersen *et al.* 2008).

The mitochondrial preparations have been characterized based on measurements of respiratory function, assays of marker enzymes for mitochondrial proteins and potential contaminants, western blotting and electron microscopy. These measurements provide detailed information on the recovery and enrichment of the mitochondria, the properties of these organelles including the intactness of the inner and outer membranes and the extent and nature of residual contamination. This has allowed the strengths and limitations of the three procedures to be identified and led to recommendations on the methods of choice for future studies.

## Materials and methods

### Animals

C57BL/6 mouse dams were purchased from Charles River Laboratories (Sulzfeld, Germany) and housed in an animal facility with a 12-h light-dark cycle (Experimental Biomedicine, University of Gothenburg). Free access to a standard laboratory chow diet (B&K, Solna, Sweden) and drinking water was provided. All animal experimentation was approved by the Ethical Committee of Gothenburg University (269-2006).

### Mitochondrial isolation

**Figure 1:**
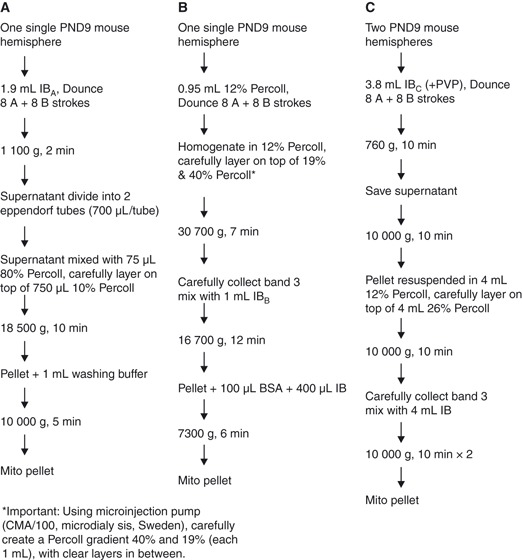
Procedure flowcharts for methods A, B and C.

On postnatal day (PND) 9, mice were killed by decapitation and the hemispheres were excised and immediately placed into ice-cold isolation buffer (IB). Brain mitochondria were isolated using discontinuous Percoll density gradient centrifugation according to methods A, B and C (Fig. 1) as described below.

Method A (Fig. 1a): one single PND9 mouse hemisphere weighing 107 ± 8 mg was dissected out and homogenized in a 2-mL all-glass Dounce tissue grinder (Kontes Glass Co., Vineland, NJ, USA) in 1.9 mL IB_A_ [225 mM mannitol, 75 mM sucrose, 1 mM EGTA, 5 mM HEPES–KOH, pH 7.2, 1 mg/mL fatty-acid-free bovine serum albumin (BSA)]. After a brief centrifugation at 1100 *g* for 2 min (4°C) (Heraeus Multifuge 3SR Plus Centrifuge, rotor no. 3332), the supernatant was divided into two Eppendorf tubes with a final volume of 700 μL each and mixed with 75 μL of freshly made 80% Percoll (80 vol% Percoll diluted in 1 M sucrose, 50 mM HEPES, 10 mM EGTA, pH 7.0), then carefully layered on the top of freshly made 10% Percoll (750 μL, 80% Percoll was diluted in IB to prepare the 10% solution) in a 2 mL Eppendorf tube and centrifuged at 18 500 *g* for 10 min (4°C). The cloudy myelin-containing top fraction was removed leaving the mitochondria-enriched pellet in the bottom of the Eppendorf tube. One microliter sucrose washing buffer [sucrose washing buffer: 250 mM sucrose, 5 mM HEPES–KOH (pH 7.2), 0.1 mM EGTA, without 1 mg/mL of BSA] was added to the pellet, mixed, and centrifuged at 10 000 *g* for 5 min at 4°C. The final mitochondrial pellet was retained on ice until used for further analysis.

Method B (Fig. 1b): one single PND9 mouse hemisphere weighing 107 ± 8 mg was dissected out and homogenized in a 2-mL all glass Dounce tissue grinder in ice cold 0.95 mL 12 vol% Percoll. Using a Microinjection pump (CMA/100. CMA/Microdialysis, Stockholm, Sweden), Percoll gradients [40% and 19%, each 1 mL; 40 vol% Percoll diluents: Trizma base 10 mM, EDTA (K) 1 mM, Sucrose 0.32 M, pH to 7.4] were carefully produced with clear layers in between (see Fig. 2 in Sims and Anderson 2008) in 6.5 mL, 16 × 64 mm Beckman Coulter polycarbonate thick wall open-top tubes (Cat. No. 355647). The homogenate was carefully added to the 40% and 19% Percoll gradient, and centrifuged in a Beckman Optima L-80 XP Ultracentrifuge, rotor 70.1 Ti (Palo Alto, CA, USA) at 30 700 *g* at 4°C for 7 min, yielding a dense band between the two lower Percoll layers (band 3). After careful removal of bands 1 and 2, band 3 (mitochondria-enriched fraction) was collected and diluted with 1 mL IB_B_ (320 mM sucrose, 1 mM EDTA, 10 mM Trizma base, pH 7.4), followed by a washing step at 16 700 g, 4°C for 12 min. After removing the supernatant, the mitochondrial pellet was transferred carefully to an Eppendorf tube containing 400 μL IB and 100 μL BSA (1 mg/mL) and mixed carefully. A last washing step using IB was performed in an Eppendorf microcentrifuge (Heraeus Multifuge 3SR Plus Centrifuge, rotor no. 3332), 7300 *g* for 6 min at 4°C, yielding a dense mitochondrial pellet. This final mitochondrial pellet was retained on ice prior to use for further analysis.

**Figure 2:**
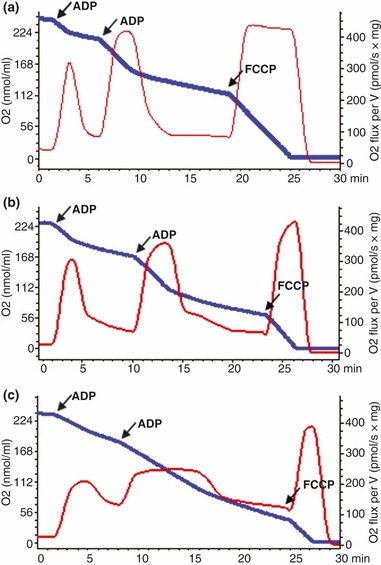
High resolution respirometry of PND9 mouse brain mitochondria isolated using methods A (a), B (b) and C (c). Experiments were performed as described in ‘Materials and methods: mitochondrial respiration’ section. The blue trace displays the oxygen concentration (nmol/mL, Y1) and the red traces show the real-time oxygen consumption rate (pmol O_2_/s × mg mitochondria, Y2) of mitochondria oxidizing 4.8 mM pyruvate and 2.5 mM malate. ADP (50 mM) was added twice to induce a transient active phosphorylating rate of respiration. Traces are representative examples of three separate experiments.

Method C (Fig. 1c): One whole brain (two hemispheres) weighing 214 ± 16 mg from a PND9 mouse were dissected out and homogenized in 3.8 mL ice-cold IB buffer with polyvinylpyrrolidone (PVP) (320 mM sucrose, 1 mM EDTA, 10 mM Trizma base, 2 mg/mL PVP, pH 7.4) using a 15 mL Dounce homogenizer with glass pestles (HDounce Tissue Grinder 15 mL, cat. no. 357544H). The homogenate were centrifuged at 4°C, 760 *g* for 10 min. Supernatant (around 3.25 mL) was saved and transferred to new tubes and centrifuged at 10 000 *g* for 10 min at 4°C using a Hettich Zentrifugen Universal 16 centrifuge. The pellet (enriched in mitochondria) was resuspended in 4 mL 12% Percoll in IB and carefully layered onto 4 mL 26% Percoll to form sharp interface between the two layers (important!) in between in Sarstedt 15 mL centrifuge tube (cat. no. 62.554.001). The gradient was centrifuged at 10 000 *g* for 10 min at 4°C. The resulting upper band and intermediate band both contained myelin and subcellular organelles. The lower band, containing most of the mitochondria, was carefully removed and put into a new tube containing 4 mL IB without PVP, centrifuged for 10 min at 10 000 *g*, 4°C and washed two times. The final mitochondrial pellet was retained on ice prior to use for further analysis.

### Mitochondrial respiration

Mitochondrial respiration was measured using a protocol modified from recently published methods (Puka-Sundvall *et al.* 2000; Frezza *et al.* 2007; Hansson *et al.* 2008; Kuznetsov *et al.* 2008) in a high-resolution respirometer, Oroboros Oxygraph-2k, with high sensitivity, low noise and concentration-dependent background correction (Oroboros Instruments, Innsbruck, Austria). Measurements were at 25°C with a final concentration of approximately 1 mg/mL mitochondria in 2.1 mL MIR05 respiration buffer [20 mM HEPES, 10 mM KH_2_ PO_4_, 110 mM sucrose, 20 mM taurine, 60 mM K-lactobionate, 0.5 mM EGTA, 3 mM MgCl_2_·6H_2_O, 1 g/L BSA (fatty acid free)]. The O_2_ flux was monitored in real-time output using DatLab 4 software. Ten microliters of 1 M pyruvate and 10 μL of 0.525 M malate were added to 2.1 mL chamber to obtain final concentrations of 4.8 and 2.5 mM, respectively. After a few minutes of recording, successive additions of 5.88 and 15 μL 50 mM adenosine diphosphate (ADP) were made to obtain a final concentration 0.14 or 0.36 mM respectively and initiate a period of phosphorylating respiration (state 3). The respiratory control ratio (RCR) was calculated as the ratio of the state 3 respiration rates following the second ADP addition to the resting respiration rate (state 4). Finally, 2.1 μL 1.0 mM carbonyl cyanide 4-trifluoromethoxy phenylhydrazone (FCCP), a mitochondrial uncoupler, was added to the chamber to give a final concentration of 1 μmol/L and induced maximum respiration.

Estimates of mitochondrial recovery were obtained as described previously (Sims 1990) based on the oxygen utilization rates measured following the addition of the second aliquot of ADP (ADP-stimulated rate) and from the maximal rate obtained following addition of FCCP (maximal uncoupled rate). Recovery was calculated by converting the measured respiration rate in the sample to a total activity for the whole isolated mitochondrial fraction (by multiplying the measured flux by the total volume in the fraction/the volume that was assayed) and expressing this as a percentage of the total activity in initial homogenates measured under the same conditions (Sims 1990).

### Mitochondrial/cytosolic marker enzymes

To measure the activity of mitochondria in the isolated mitochondrial fractions, as well as for detecting the cytosolic contaminations, the following mitochondrial/cytosolic marker enzymes were used. Cytosolic enzyme lactate dehydrogenase (LDH; Roche, Boehringer Mannheim, Germany; Cat. No. 1644793) was used to measure contamination with synaptosomes or other structures such as gliosomes; Citrate Synthase Assay (Citrate Synthase Assay Kit Sigma, Gothenburg, Sweden; Cat. No. CS0720) was used as an exclusive marker of the mitochondrial matrix. It was also used for testing the intactness of the mitochondrial inner membrane because the citrate synthase activity is not readily measurable in intact mitochondria owing to the impermeability of the inner membrane to the substrates. The recovery of citrate synthase was calculated as the percentage of total citrate synthase activity in the mitochondrial preparation compared with that in the initial homogenate. Percentage of the rupture of mitochondria was calculated from the ratio between the citrate synthase activities in the mitochondrial preparation in Bicine Buffer (a buffer containing no detergent) versus the activity in the same mitochondrial preparation treated with Cell Lysis Reagent (ruptured mitochondria). The activity observed in the sample suspended in the Cell Lysis Reagent represents the total citrate synthase activity present in the mitochondria, because the mitochondria in this sample are ruptured and the substrate in the reaction mixture is readily available to the enzyme. The activity of the citrate synthase observed in the sample suspended in Bicine Buffer represents the activity resulting from broken mitochondria in the isolated mitochondria preparation.

Cytochrome c oxidase activity (Assay kit Cat. No. KC310100 from BioChain Institute, Inc., Hayward, USA) was measured as a further marker of mitochondrial activity and of the integrity of mitochondrial outer membrane. The integrity of the outer membrane was assessed by measuring cytochrome c oxidase activity in mitochondrial membrane in the presence and absence of the detergent, *n*-dodecyl β-d-maltoside. Cytochrome *c* oxidase locates in the inner membrane of the mitochondria. Cytochrome *c* cannot access the cytochrome *c* oxidase when the outer membrane is intact. So the ratio between activity without and with *n*-dodecyl β-d-maltoside presence is a measure of the integrity of the mitochondrial outer membrane. *n*-Dodecyl β-d-maltoside is one of the few detergents that maintain the cytochrome c oxidase dimer in solution at low detergent concentration (Musatov *et al.* 2000), therefore maintaining the enzyme activity intact. So the ratio between activity without and with *n*-dodecyl β-d-maltoside presence is a measure of the integrity of the mitochondrial outer membrane. The measurements were carried out according to the manufacturer’s instructions.

### Electron microscopy

Electron microscopy (EM) was performed as previously described (Wang *et al.* 2009). In brief, after the isolation, brain mitochondria from PND9 mice (*n* = 3/method) were fixed with a mixture of 2% paraformaldehyde, 2.5% glutaraldehyde, and 0.02% sodium azide in 0.05 M sodium cacodylate, embedded in epoxy resin (Agar 100). The preparations were sectioned and stained with lead citrate and uranyl acetate and examined with a Zeiss 912AB electron microscope equipped with a MegaView III camera (Soft Imaging Systems, Münster, Germany) for digital image capture. The proportion of free mitochondria was determined by examining 250 particles and organelles in low magnification EM pictures. Free mitochondria were defined as double membrane ribbed structures that were distinguishable from other parts of the tissue subfractions such as vesicles and cell membranes which lacked the clear double membrane structure.

### Western blotting

Western blotting was performed as previously described (Wang *et al.* 2003). Briefly, after blocking with 30 mM Tris–HCl (pH 7.5), 100 mM NaCl and 0.1% Tween 20 (TBS-T) containing 5% fat-free milk powder for 1 h at 25°C, the membranes were incubated with primary antibodies: anti-Lamin B (1 : 200, 1 μg/mL, sc-6217, goat polyclonal antibody; Santa Cruz Biotechnology, Santa Cruz, CA, USA), anti-synapsin Ia/b (N-19) (1 : 500, 0.4 μg/mL, sc-7379, goat polyclonal antibody; Santa Cruz), anti-OxPhos Complex I (1 : 1000, 1 μg/mL, A21344, clone 20C11; Molecular Probe, Eugene, OR, USA) and anti-2′,3′-cyclic nucleotide 3′ phosphodiesterase (CNPase, 1 : 200, MS-349-P; Lab Vision, Fremont, CA, USA). After washing, the membranes were incubated with a peroxidase-labeled secondary antibody for 30 min at 25°C (horse anti-goat 1 : 2000 or horse anti-mouse 1 : 4000). Immunoreactive species were visualized using the Super Signal West Dura substrate (Pierce, Rockford, IL, USA) and a LAS 3000 cooled CCD camera (Fujifilm, Tokyo, Japan).

## Results

### The mitochondrial respiratory properties

The yield of total mitochondrial protein from one single PND9 mouse hemisphere was: 3.89 ± 0.14, 3.59 ± 0.65, and 8.59 ± 0.21 μg/mg wet tissue for methods A, B and C, respectively. The respiratory control ratio RCR (state 3 respiration/state 4 respiration) is commonly used as an indicator of the integrity/functionality of isolated mitochondria. Figure 2 shows representative traces for oxygen utilization for the fractions isolated using each of the three procedures. The average values for respiratory activities and the respiratory control ratios are presented in Table 1. Average values for this ratio were 5.0 ± 0.2 for method A, 4.9 ± 0.5 for method B and 2.8 ± 0.4 for method C (Table 1, Fig. 2). Methods A and B generated a similar degree of mitochondrial recovery. This was only a little lower than recoveries of approximately 10% that have been reported for adult rat when whole forebrain was used as starting material (Sims and Anderson 2008). Method C resulted in fractions with less proportion of mitochondria with respiratory activity.

**Table 1:** Respiratory properties of PND9 mouse brain mitochondria assessed with 5 mM pyruvate and 2.5 mM malate as metabolic substrates

	Respiratory activity			RCR (state 3/state 4)
State 3	State 4	Uncoupled
Method A	244 ± 55	48 ± 8	245 ± 50	5.0 ± 0.2
Method B	242 ± 42	50 ± 12	258 ± 32	4.9 ± 0.5
Method C	185 ± 58	67 ± 20	239 ± 40	2.8 ± 0.4

Results are presented as mean ± SD.

Respiratory activity is shown in nmol O_2_ consumed/min/mg mitochondrial protein.

*n *= 3 for each method.

### Mitochondria activity and integrity of inner and outer membrane

Estimates of the recovery of mitochondria for each of the methods based on their respiratory activities are presented in Table 2. Recovery of the maximal uncoupled activity was calculated as the percentage of the maximum respiration rates following addition of FCCP in the mitochondria to that in the initial homogenate. ADP-stimulated activity was calculated as the percentage of the state 3 respiration rates following the second ADP addition in the mitochondria to that in the initial homogenate. Therefore the recovery of maximal uncoupled oxygen utilization provides a measure of the proportion of metabolically active mitochondria in the isolated fraction relative to the total number of mitochondria (free and synaptosomal mitochondria) in the initial homogenate. The values calculated from total ADP-stimulated respiratory activity give a measure of the recovery of free (non-synaptosomal) mitochondria in the final preparation compared with free mitochondria in the initial homogenate fraction. This does not include mitochondria in synaptosomes and other membrane-enclosed structures because of the inability of ADP to cross-intact plasma membranes. These recoveries could potentially be affected both by losses of mitochondria during subfractionation and by a decrease in respiratory activity of the recovered organelles induced during their isolation.

**Table 2:** Recovery (percentage of initial homogenate) of mitochondrial respiratory activity (oxygen utilization) in the mitochondrial fraction isolated from PND9 mouse forebrain

	Recovery (% of initial homogenate activity)	
Maximal uncoupled activity	ADP-stimulated activity
Method A	6.7 ± 1.1	9.2 ± 1.3
Method B	6.5 ± 0.9	8.8 ± 2.3
Method C	8.5 ± 2.9	7.4 ± 1.4

Results are presented as mean ± SD.

*n *= 3 for each method.

Besides, method A and B generated fractions with high citrate synthase activity indicating mitochondria were well preserved with the isolation methods used. Method C was inferior with a lower recovery of citrate synthase (Table 3).

**Table 3:** Activity and recovery of enzyme marker citrate synthase in the mitochondrial fraction isolated from PND9 mouse forebrain

	Specific activity	Recovery (% of initial homogenate)	% rupture of Mitochondria
Method A	183 ± 42	6.5 ± 2.7	13.7 ± 1.1
Method B	270 ± 63	5.2 ± 3.8	28.3 ± 10.0
Method C	159 ± 9	2.3 ± 0.4	12.6 ± 1.3

Results are presented as mean ± SD.

Unit for citrate synthase is μmol/mL/min/μg prot.

*n *= 4–6 for each method.

To examine the intactness of the outer mitochondrial membrane, we measured the mitochondria-specific cytochrome c oxidase activity in the isolated mitochondria and the degree of mitochondrial integrity was presented as a percentage of mitochondria with intact mitochondrial outer membrane. All three methods yielded isolated mitochondria with high degree of outer mitochondrial membrane integrity (97.8 ± 1.7, 95.4 ± 2.1, 97.3 ± 1.3 for Method A, B and C respectively).

### Electron microscopic evaluation

**Figure 3:**
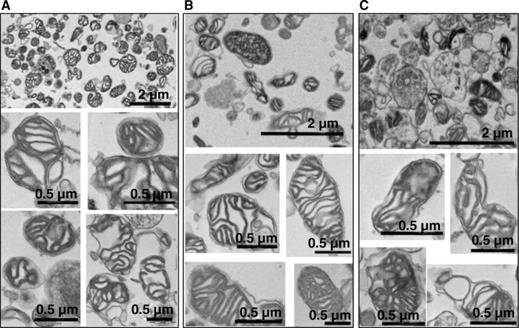
Electron photomicrographs of mitochondria from methods A, B and C. Bar = 2 μm in low magnification pictures, Bar = 0.5 μm in all high magnification pictures.

Electron microscopic evaluation revealed substantial differences in material yield and composition after the three preparation procedures (Fig. 3). Methods A and B produced the most pronounced enrichment of mitochondria. After isolation using method C, less free mitochondria were obtained together with a larger contamination of subcellular vesicles of different identity. A total of 250 organelles and particles from random fields of low magnification view were counted and the number of free mitochondria (expressed as % of all organelles/particles) amounted to 62.5%, 66.8% and 25.5% for methods A, B and C, respectively. In the mitochondrial pellet analyzed under EM, the other organelles such as synaptosomes and nuclei, as well as indefinable material such as small spheres (possibility of contamination be residual Percoll particles) were visible as well.

Our EM analysis of mitochondria revealed that mitochondria isolated from the neonatal brain possessed well developed cristae (Fig. 3). Moreover, their crista oriented in parallel, perpendicularly or obliquely to the mitochondrial long axis. Some of the cristae were transverse the entire width of mitochondria. But in general the mitochondrial matrix compartment was slightly osmiophilic-like, that is, a ‘condensed’ appearance (Fig. 3) after the isolation.

### Subcellular contaminations

**Figure 4:**
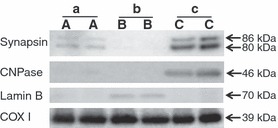
Representative western blots showing the contamination by other cellular fractions in the isolated mitochondria from PND 9 mouse brain using different protocol A (a), protocol B (b) and protocol C (c). Synapsin, synapse marker; CNPase, myelination marker; Lamin B, nuclear marker; COX I, loading control.

Most of the contamination from synaptosomes, nuclear and myelin should be eliminated during the preparation. Lactate dehydrogenase is a cytosolic enzyme that is predominantly associated with contaminating synaptosomes in these preparations and CNPase is a myelin-associated enzyme. Method B produced the most purified mitochondria based on the low recovery of the cytosolic enzyme marker LDH (Table 4), and the low residual content of the synaptosome marker synapsin, nuclear marker lamin B and myelin marker CNPase (Fig. 4). Method A had slightly higher contamination of synaptosomes compared with method B (Fig. 4) as well as a relatively higher recovery of the enzyme marker LDH (Table 5). Method C produced mitochondrial isolates with a reasonably low LDH recovery, but suffered from heavy contamination with both myelin and synaptosomes (Fig. 4).

**Table 4:** Activity and recovery of enzyme marker LDH in the mitochondrial fraction isolated from PND9 mouse forebrain

	Specific activity	Recovery (% of initial homogenate)
Method A	0.8 ± 0.1	1.0 ± 0.3
Method B	0.7 ± 0.1	0.6 ± 0.2
Method C	1.2 ± 0.1	1.5 ± 0.7

Results are presented as mean ± SD.

Unit for LDH-specific activity is units/mL (in the same protein concentration for all three methods).

*n *= 4–6 for each method.

**Table 5:** Summary of the performances of the three isolation methods

	Method A	Method B	Method C
Instrument requirement	General purpose benchtop centrifuge	High-speed centrifuge	General purpose benchtop centrifuge
Process length	60 min	90 min	60 min
EM	Mitochondria enriched, mitochondria morphology intact	Mitochondria enriched, mitochondria morphology intact	Mitochondria enriched and mitochondria morphology intact, cellular contamination easy to see
Cytochrome *c* oxidase (% integrity of outer membrane)	97.8 ± 1.7	95.4 ± 2.1	97.3 ± 1.3
Myelin contamination	No	No	Yes
Synapse contamination	Low	No	High
Nuclear contamination	No	Low	No

## Discussion

Mitochondria are key regulators of death in most cells in species ranging from C elegans to mammals (Zhivotovsky *et al.* 2009). They play a major role in necrosis, apoptosis and autophagic cell death (Tasdemir *et al.* 2008; Galluzzi *et al.* 2009) and several recent reviews provide detailed information on the general mechanisms involved (Kroemer *et al.* 2007). Furthermore, mitochondria are implicated in most degenerative diseases in the CNS (Schapira 2008). In the immature brain, NMDA/AMPA receptor activation, increased intracellular Ca^2+^ and accumulation of reactive oxygen species exert stress on mitochondria after HI. Indeed, blockade of NMDA receptors improves mitochondrial respiration and reduces injury (Gilland and Hagberg 1996; Gilland *et al.* 1998a,b). These upstream events combine with increased pro- *vs.* anti-apoptotic B cell lymphoma 2 (Bcl-2) family protein balance (Northington *et al.* 2001; Ness *et al.* 2006) and inflammatory activation of death receptors (Felderhoff-Mueser *et al.* 2000), to induce mitochondrial permeabilization. This, leads to release of cytochrome *c* and apoptosis-inducing factor resulting in activation of caspase- (Wang *et al.* 2001) and non-caspase-dependent (Zhu *et al.* 2007) pathways, further impaired respiratory and Ca^2+^ regulatory capacity and ultimately to cell death. For gaining comprehensive understandings on the role of mitochondria in the immature brain injury, it is essential to have a good mitochondrial isolation method specifically for the immature mouse brain.

As recently summarized, isolation procedures for brain mitochondria for adult mice/rats have been developed (Sims and Anderson 2008). For the immature brain, a few methods have been used (Rajapakse *et al.* 2001; Caspersen *et al.* 2008) but the relative merits of these techniques are unknown. The present study gives information on the properties of the mitochondria isolated by these procedures providing a basis for selecting the procedure best suited to the particular purposes of a study. Furthermore, the current study indicates that mitochondrial isolation procedures have to be adapted according to developmental age.

An ideal isolation method for brain mitochondria would generate fractions containing mitochondria with intact morphology, high respiratory activity, preserved integrity of both the mitochondrial inner and outer membrane and no contamination with other cell fractions such as synaptosomes, myelin, and nuclei. We found that both method A and B produced mitochondria with high activity and intact mitochondrial inner and outer membranes, and relatively low contamination with other cellular components, as summarized in Table 5. Therefore, both represent satisfactory methods for isolation of neonatal mouse brain mitochondria. Method A is a relatively simple and fast isolation procedure, and offers a slightly higher yield of mitochondria using the same amount of brain material compared with method B. Methods A and C are both easy to perform and require relatively short times for the whole preparation process. Method B requires high speed centrifugation that might not be available for all laboratories (Table 5) and the isolation process is more complex than methods A and C. One critical step for all three methods is to create clear layers between different concentrations of Percoll gradient. The other important components in mitochondrial isolation have been discussed thoroughly in a recent publication (Sims and Anderson 2008).

All three of the procedures make use of discontinuous Percoll gradient centrifugation as the key step for isolating an enriched mitochondrial fraction but differ markedly in the concentration profile of this gradient. Furthermore, the methods as used in the present study differ in the use of additional centrifugation steps for subfractionation. In method B, the initial crude homogenate was directly fractionated using density-gradient centrifugation whereas method A employed an initial slow-speed centrifugation to reduce contamination with nuclei and cell debris. Method C similarly used an initial slow speed centrifugation step but also includes a medium speed centrifugation to generate a crude mitochondrial fraction prior to further fractionation. The procedures also differed in other details including the amount of starting material that was fractionated and the composition of solutions used during mitochondrial isolation.

Figure 2 shows representative traces for oxygen utilization for the fractions isolated using each of the three procedures. The average values for respiratory activities and the respiratory control ratios are presented in Table 1. Average values for this ratio were 5.0 ± 0.2 for method A, 4.9 ± 0.5 for method B and 2.8 ± 0.4 for method C (Table 1, Fig. 2). Methods A and B generated a similar degree of mitochondrial recovery.

According to the EM results, the mitochondria obtained from method A was rather similar to the mitochondria obtained from the previously reported method used for adult rat brain (Sims 1990; Hansson *et al.* 2003) and neonatal mouse brain (Caspersen *et al.* 2008). Using all three methods, mitochondria were enriched and had preserved morphology, in comparison with previous results in adult and neonatal brain mitochondria (Sims 1990; Hansson *et al.* 2003; Caspersen *et al.* 2008). Among all three methods, method C suffered from the most pronounced contamination and method B least contamination.

The RCR ratio in method A and B are comparable with the previously published mitochondrial isolation method range 5.4–6.2 for adult rat brain mitochondrial isolation (Sims and Anderson 2008) and 4.2 ± 1.8 for neonatal rat brain mitochondrial isolation (Rajapakse *et al.* 2001). Therefore, methods A and B both produced mitochondria with good integrity. According to the judgment by enzyme marker citrate synthase and cytochrome *c* oxidase measurement, all three methods produced mitochondria with satisfactory mitochondrial activity and integrity of inner/outer membrane.

EM is a very useful method to evaluate the results of mitochondrial isolation from different preparations. In the present study, EM pictures showed that in general the isolated mitochondria from all three methods exhibited a slightly altered morphology as compared with the appearance of the organelle in its intracellular location but comparable to the morphology after previously published isolation procedures for adult and neonatal rat brains (Sims 1990; Hansson *et al.* 2003; Caspersen *et al.* 2008).

Cellular contamination is abundant in method C for both synapsin and myelin, while method B proved to be the method that produces the purest mitochondrial isolates, as very limited nuclear contamination was detected. Method A has some synapsin and LDH contamination but seems to be acceptable for most purposes.

In conclusion, among all three methods tested, methods A and B are both suitable methods for neonatal brain mitochondrial isolation. The method of choice depends on the purpose and nature of the particular study undertaken. Method A offers the advantage of being more rapid and producing more mitochondria yields from the same amount of tissue making it useful for routine applications; however, if it is critical to obtain isolates with less contamination with synaptosomes and other subcellular components cytosolic/synaptosomal contamination, method B would be preferable.

## Abbreviations

ADP: adenosine diphosphate
BSA: bovine serum albumin
CNPase: 2′,3′-cyclic nucleotide 3′ phosphodiesterase
EM: electron microscopy
FCCP: carbonyl cyanide 4-trifluoromethoxy phenylhydrazone
IB: isolation buffer
LDH: lactate dehydrogenase
PND: postnatal day
PVP: polyvinylpyrrolidone
RCR: respiratory control ratio
